# Drug-Eluting versus Bare-Metal Stent for Treatment of Saphenous Vein Grafts: A Meta-Analysis

**DOI:** 10.1371/journal.pone.0011040

**Published:** 2010-06-10

**Authors:** Pascal Meier, Emmanouil S. Brilakis, Roberto Corti, Guido Knapp, Mehdi H. Shishehbor, Hitinder S. Gurm

**Affiliations:** 1 University of Michigan Medical Center, Ann Arbor, Michigan, United States of America; 2 Veterans Affairs Ann Arbor Healthcare System, Ann Arbor, Michigan, United States of America; 3 Division of Cardiovascular Diseases, Veterans Affairs North Texas Healthcare System, Dallas, Texas, United States of America; 4 Department of Cardiology, University Hospital Zurich, Zurich, Switzerland; 5 Department of Statistics, TU Dortmund University, Dortmund, Germany; 6 Cardiovascular Medicine, Cleveland Clinic Foundation, Cleveland, Ohio, United States of America; Copenhagen University Hospital, Denmark

## Abstract

**Background:**

Saphenous vein grafts develop an aggressive atherosclerotic process and the efficacy of drug eluting stents (DES) in treating saphenous vein graft (SVG) lesions has not been convincingly demonstrated. The aim of this study was to review and analyze the current literature for controlled studies comparing DES versus bare metal stents (BMS) for treatment of SVG stenoses.

**Methodology/Principal Findings:**

We searched several scientific databases and conference proceedings up to March 15, 2010 for controlled studies comparing target vessel revascularization (TVR) between DES and BMS. Summary odds ratios (OR) for the primary endpoint TVR and secondary endpoints infarction, stent thrombosis and death were calculated using random-effect models. A total of 29 studies (3 randomized controlled trials RCT) involving 7549 (202 in RCT) patients were included. The need for target vessel revascularization in the DES group tended to be lower compared to BMS for the 3 RCT (OR 0.50 [0.24–1.00]; p = 0.051) and for observational studies (0.62 [0.49–0.79]; p<0.001). There was no significant difference in the risk for myocardial infarction in the RCT (OR 1.25 [0.22–6.99]; p = 0.250) but a lower risk for DES based on the observational studies 0.68 [0.49–0.95]; p = 0.023. The risk for stent thrombosis was found to be non-different in the RCT (OR 0.78 [0.03–21.73], p = 0.885) while it was in favor of DES in the observational studies (0.58 [0.38 – 0.84]; p<0.001). The mortality was not significantly different between DES and BMS in the RCT's (OR 2.22 [0.17 – 29.50]; p = 0.546) while the observation studies showed a decreased mortality in the DES group (0.69 [0.55–0.85]; p<0.001).

**Conclusion:**

DES may decrease TVR rate in treatment of SVG stenoses. No differences in reinfarction rate, stent thrombosis or mortality was found between the DES and BMS groups in the RCT's while the observational data showed lower risk for myocardial infarction, stent thrombosis and death in the DES group. This may be a result of patient selection bias in the observational studies or represent a true finding that was not the detected in the RCT analysis due to limited statistical power.

## Introduction

Coronary artery bypass graft (CABG) is among the most frequently performed surgical procedures in the U.S. and Europe and a mainstay of therapy for coronary artery disease (CAD). Saphenous vein grafts are the most common type of the grafts used in coronary by-pass surgery. SVG interventions currently account for about 5–10% of total percutaneous coronary interventions (PCI) annually in the United States.[1], [2] This number is likely to increase in the near future since there is emerging evidence that even lower degree stenoses (30–60%) may profit from stent implantations;[3] very much in contrast to stenoses in native vessels where increasing data suggest that only hemodynamically significant higher degree stenoses should be treated.[4] The natural and post-interventional biological behaviour of saphenous vein grafts clearly differs from native vessels, they are at higher risk for restenosis.[5] While BMS are currently the gold standard for SVG stenosis, the off-label use of DES has shown promising results in several observational studies while there is a dearth of adequately powered randomized trials. [6], [7], [8] These trials have produced conflicting results and were rather small.

While DES have demonstrated superiority regarding TVR in treatment of native coronary arteries, saphenous vein graft stenting is an entity that has to be investigated specifically. SVG are different in many regards from arterial vessels. Media layers of the SVG are thinner than that of coronary arteries, and thus, are more susceptible to mechanical damage by stents and balloon pressure. Media fracture has been associated with exaggerated neointimal response.[9] Usually, degenerated vein grafts stenoses consist of soft friable plaques without fibrous cap. Classical atherogenesis in contrast probably plays a minor role here. Instead, hypothesized mechanisms are intimal thrombus formation that converts into fibrous plaque, change in wall stress (“arterialization” of the vein) and impairment of intrinsic vascular supply.[10], [11] This intimal hyperplasia in the first months after surgery represents the substrate on which coronary atherosclerosis develops.

The aim of this meta-analysis was to systematically review the current literature for controlled randomized and non-randomized studies comparing drug-eluting stents (DES) versus bare-metal stents (BMS) for treatment of SVG stenoses with a primary focus on need for re-intervention. Further endpoints of interest were mortality, stent thrombosis and myocardial infarction.

## Methods

### Eligibility criteria

Planning and study design was done by two authors (HSG, PM) including creation of an electronic database with variables of interest (Microsoft Excel). Primary and secondary endpoints, variables of interest and search strategy (databases, sources for unpublished data) were defined in a strategy outline (File S1).

We included controlled (randomized and non-randomized) studies that compared DES and BMS (with and without the use of protection devices) in patients with saphenous vein graft (SVG) stenosis. The outcome of primary interest was TVR and the secondary outcomes were myocardial infarction, stent thrombosis or death. Because we expected paucity of data, observational studies were not excluded *a-priori* even though the primary focus was on RCT.

We searched EMBASE, MEDLINE, the Cochrane Central Register of Controlled Trials, International Pharmaceutical Abstracts database, ISI Web of Science, and google scholar from 2002 through March 15, 2010. In addition, abstract lists and conference proceedings from the 2006 to 2010 scientific meetings of the American College of Cardiology, and the 2006 to 2009 meetings of the European Society of Cardiology, the Transcatheter Cardiovascular Therapeutics, and the American Heart Association were included. We also considered published review articles, editorials, and internet-based sources of information (www.tctmd.com, www.theheart.org) to assess potential information on studies of interest.

Search strategy for MEDLINE was: “saphenous vein graft” [All Fields] AND (“bare-metal stent” [All Fields] OR “drug-eluting stent” [All Fields] OR “paclitaxel-eluting stent”[All Fields] OR “sirolimus-eluting stent” ”[All Fields] OR “everolimus-eluting stent” [All Fields] OR zatarolimus-eluting stent” [All Fields] OR “stents” [MeSH Terms]). No restriction on subheadings was applied. Similar but adapted search terms were used for the other literature databases.

Reference lists of selected articles were reviewed for other potentially relevant citations. Authors of selected studies were contacted to obtain further information. All trials comparing DES versus BMS in patients with SVG were included in this analysis.

### Study selection

**Figure 1:**
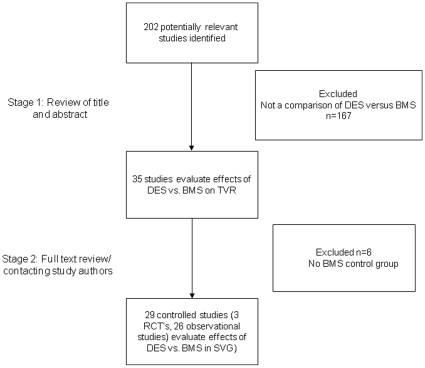
Flow chart depicting outline of the search and selection strategy. DES = drug-eluting stent; BMS = bare metal stent; SVG = saphenous vein graft.

In a two-step selection process, two investigators (HSG and PM) independently reviewed the titles and abstracts of all citations to identify all potentially relevant studies. In a second step the corresponding publications were reviewed in full text by the same two investigators to assess if studies were meeting the following inclusion criteria: direct comparison of DES vs. BMS, controlled trial including a BMS control group, and reporting clinical outcomes (TVR, death, ST or MI; Figure 1). Reviewers were not blinded to study authors or outcomes. Final inclusion of studies was based on agreement of both reviewers.

### Data extraction

The relevant information from the articles including baseline clinical characteristics of the study population was extracted by two investigators (PM and HSG) into an electronic database. Extracted data were compared and in case of disagreement original data were re-checked by both investigators. Where data on the primary endpoint could not be extracted from the publication, corresponding authors were contacted. We extracted data on the primary endpoint target vessel revascularization (or target lesion revascularization alternatively), infarction, stent thrombosis and mortality. We also extracted data on important co-variables (follow up time, mean age of patients, type of drug-eluting stents used, use of distal embolic protection device, age of graft).

### Data synthesis and analysis

All analyses were performed on an intention-to-treat basis. Continuity correction was used when an event did not occur in one group.[12]We evaluated the presence of heterogeneity across trials with the I^2^ statistics. Observational studies and RCT were combined separately and pooled odds ratios (OR) of effect sizes for DES compared with BMS were estimated using random-effect models with the DerSimonian-Laird approach. Publication bias was evaluated based on the RCT with the Egger's test and visually with a funnel plot.[13] For randomized trials, only data from peer-reviewed publications were used to be able to assess study quality, proper randomization etc. Published and unpublished data have been used for observational data, these data are used as a secondary confirmatory analysis. The quality of each RCT and the risk for bias in the individual RCT was assessed by two investigators (HSG, PM) based on the Jadad scale [14]. The score was used to ensure sufficient quality but was not implemented in the analyses because of significant limitations of such approaches.[14], [15] Observational studies are at risk for selection bias and therefore, we did not mix randomized and non-randomized data but present the non-randomized data as a secondary confirmatory analysis. A sensitivity analysis with updated unpublished data from the randomized trials that has been presented at scientific meetings has been performed. To evaluate for explanations for heterogeneity of study results, the influence of the following factors was evaluated by stratified analyses: type of DES used in the DES group, publication date, study size, duration of follow up.

Weighted meta-analytical prevalence estimates for outcome in DES and BMS patients were calculated using the variance stabilising Freeman-Tukey double arcsine transformation with an inverse variance random effects model.[16]All analyses were performed with R version 2.9.0[17] (packages “meta”, “metafor” and “rmeta”) and SAS, version 9.2 (SAS Institute, Cary, NC) (proc mixed).[18] Data for odds ratio and prevalence estimates will be presented as point estimates followed by 95% confidence interval estimates in square brackets.

## Results

A total of 202 articles were reviewed, and 29 studies including 7549 patients, satisfied the predetermined strict inclusion criteria; of those, 3692 were treated with a BMS and 3857 with a DES. A subset of 202 patients were randomly assigned to BMS or DES in an RCT (Figure 1). [6], [7], [8], [19] One randomized trial is a subgroup analysis of a larger trial.[7] Tables 1, 2 and 3 summarize the characteristics of the studies. The other 26 studies (n = 7347) were observational registries.[20], [21], [22], [23], [24], [25], [26], [27], [28], [29], [30], [31], [32], [33], [34], [35], [36], [37], [38], [39], [40], [41], [42], [43]. All trials included in this analysis had sufficient quality and all 3 trials were included in the analysis (Table S1).

**Table 1:** Characteristics of included randomized trials.

Study	N	Stent	Follow up (mts)	Remarks	Patient age (yrs)	Graft age (yrs)	Protection device (%)
BASKET	13	BMS	18		71	na	na
	34	DES	18	SES and PES	71	na	na
Delayed RRISC	37	BMS	median 32		72	12.6	na
	38	DES	median 30.5	SES	73	12.4	na
SOS	39	BMS	median 18		67	12.0	56
	41	DES	median 18	PES	66	11.0	51

BMS: bare-metal stent;

DES: drug-eluting stent;

na: not available;

PES: paclitaxel-eluting stent;

SES: sirolimus-eluting stent;

TLR: target lesion revascularization;

TVR: target vessel revascularization.

**Table 2:** Characteristics of included observational studies.

Study	N	Stent	Follow up (mts)	Remarks	Patient age (yrs)	Graft age (yrs)	Protection device (%)
Ge et al.	89	BMS	6	na	67	9.2	22.5
	61	DES		na	67	9.7	31.1
Lee et al.	84	BMS	mean 9		69	na	15
	139	DES	mean 10	211 SES; 78 PES	69	na	19
Chu et al.	57	BMS	12		71	9.4	100
	48	DES	12	SES	69	10.1	100
Hoffman et al.	60	BMS	6 (TLR)		67	na	52
	60	DES	6 (TLR)	PES	67	na	64
Wohrle et al.	26	BMS	12		70	9.1	0
	13	DES	12	PES	71	11.4	0
Ellis et al.	175	BMS	12		69	9.8	25.1
	175	DES	12	SES	70	10.0	35.6
Minutello et al.	50	BMS	mean 20		69	na	48
	59	DES	mean 21	SES	71	na	71.2
Bansal et al.	72	BMS	mean 33		65	na	27
	37	DES	mean 34	95% SES; 5% PES	68	na	39
Gioia et al.	119	BMS	up to 23		70	11.0	na
	106	DES	up to 23	106 SES; 48 PES	71	11.0	na
Assali et al.	43	BMS	24		71	11.4	48
	68	DES	24	SES	70	10.8	38
van Twisk et al.	128	BMS	48		69	na	na
	122	DES	48	SES, PES	68		na
Vignali et al.	288	BMS	median 13.7		71	10.7	na
	72	DES	median 13.8	na	75	9.0	na
Wilson et al.	281	BMS	9		na	na	na
	418	DES	9	243 SES, 171 PES	na	na	na
May et al.	176	BMS	12 (TLR)		69	na	na
	201	DES	13 (TLR)	na	69	na	na
Voudris et al.	40	BMS	mean 22.5		na	na	na
	43	DES	mean 22.6	90% SES; 10% PES	na	na	na

BMS: bare-metal stent;

DES: drug-eluting stent;

na: not available;

PES: paclitaxel-eluting stent;

SES: sirolimus-eluting stent;

TLR: target lesion revascularization;

TVR: target vessel revascularization.

**Table 3:** Characteristics of included observational studies (continued).

Study	N	Stent	Follow up (mts)	Remarks	Patient age (yrs)	Graft age (yrs)	Protection device (%)
Moore et al.	173	BMS	12 (TLR)		67	na	na
	171	DES	13 (TLR)	SES,PES	69	na	na
Okabe et al.	344	BMS	12		70	na	21
	138	DES	12	17 SES; 66 PES	70	na	26
Applegate et al.	74	BMS	24		69	na	47
	74	DES	24	67 SES; 7 PES	69	na	53
Shishehbor et al.	349	BMS	35		69	na	30
	217	DES	35	na	70	na	56
Lozano et al.	114	BMS	30		71	121	na
	98	DES	30	na	66	108	na
Brodie et al.	343	BMS	9		69	na	33.7
	785	DES	9	59% SES, 38% PES, 3% both	68	na	37.3
Ramana et al.	170	BMS	mean 34		69.1	12.9	na
	141	DES	mean 34	100% SES	70	11.5	na
Kaplan et al.	33	BMS	12		70.5	7.6	na
	37	DES	12	100% SES	72.3	7.5	na
Jin-cheng et al.	47	BMS	12		71	na	31
	50	DES	12	mixed	74	na	30
Goswami et al.	95	BMS	36 (TLR)		69.5	na	na
	284	DES	36 (TLR)	84% SES, 16% PES	70.7	na	na
Latib et al.	174	BMS	24		na	na	na
	127	DES	24	na	na	na	na

BMS: bare-metal stent;

DES: drug-eluting stent;

na: not available;

PES: paclitaxel-eluting stent;

SES: sirolimus-eluting stent;

TLR: target lesion revascularization;

TVR: target vessel revascularization.

### Primary endpoint

#### TVR

**Figure 2:**
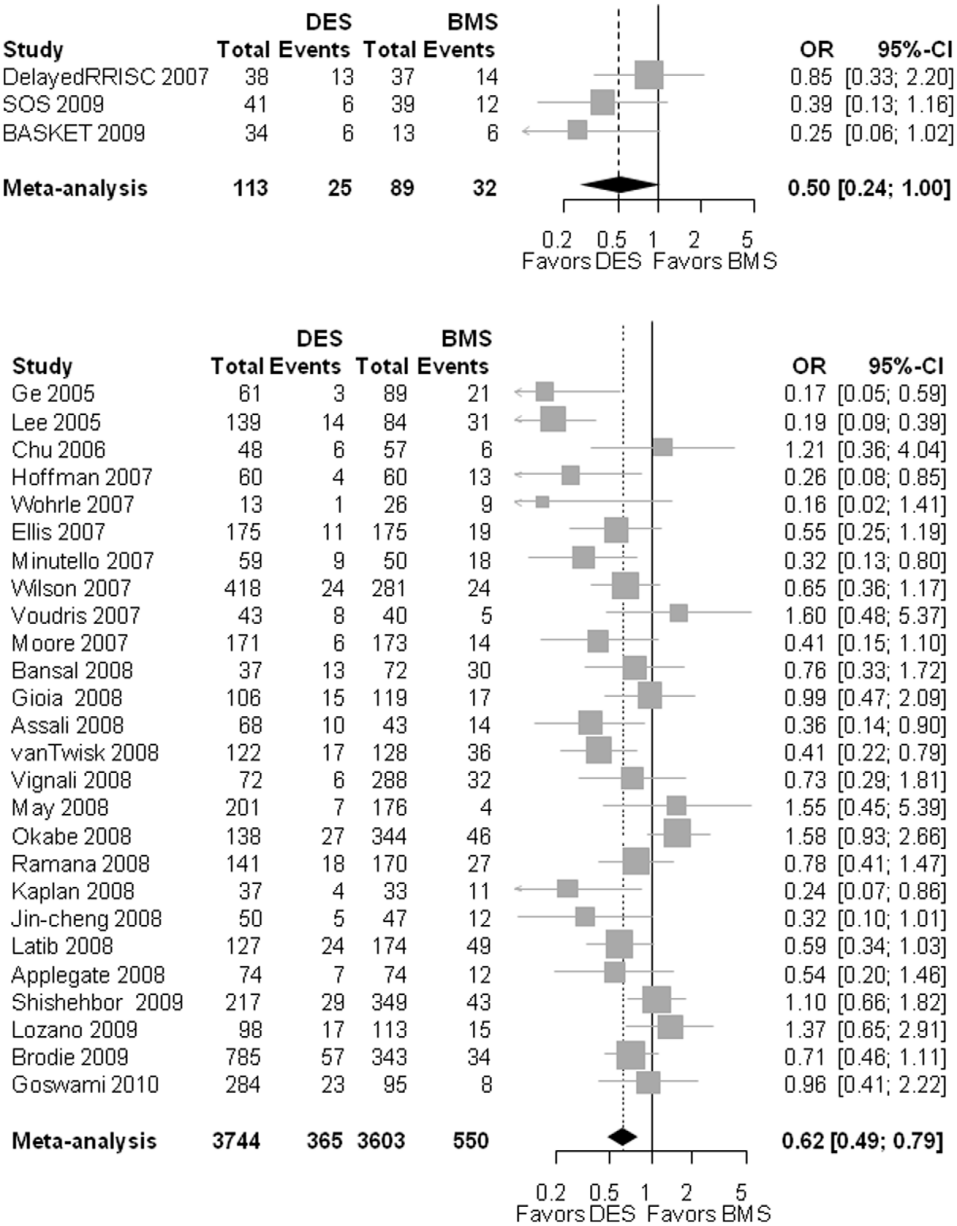
The Forest plot of odds ratios (OR) of target-vessel revascularization (TVR). Sizes of data markers are propo rtional to the weight of each study in the meta-analysis. Horizontal bars, 95% confidence intervals (CI). Observational = observational, non-randomized controlled studies; DES = drug-eluting stent; BMS = bare metal stent; RCT = randomized controlled trials.

In the 3 RCT, TVR occurred in 22.3% [12.1 – 34.7%] of patients with DES and in 36.3% [26.9–46.4%] of patients with BMS. The summary OR was 0.50 [0.24–1.00]; p = 0.051; heterogeneity I^2^ = 16.2%; p = 0.303; Figure 2) in favor of DES.

The OR for the observational studies was 0.62 [0.49–0.79]; p<0.001; heterogeneity: I^2^ = 56.3%, p<0.001).

### Secondary endpoints

#### Myocardial infarction

**Figure 3:**
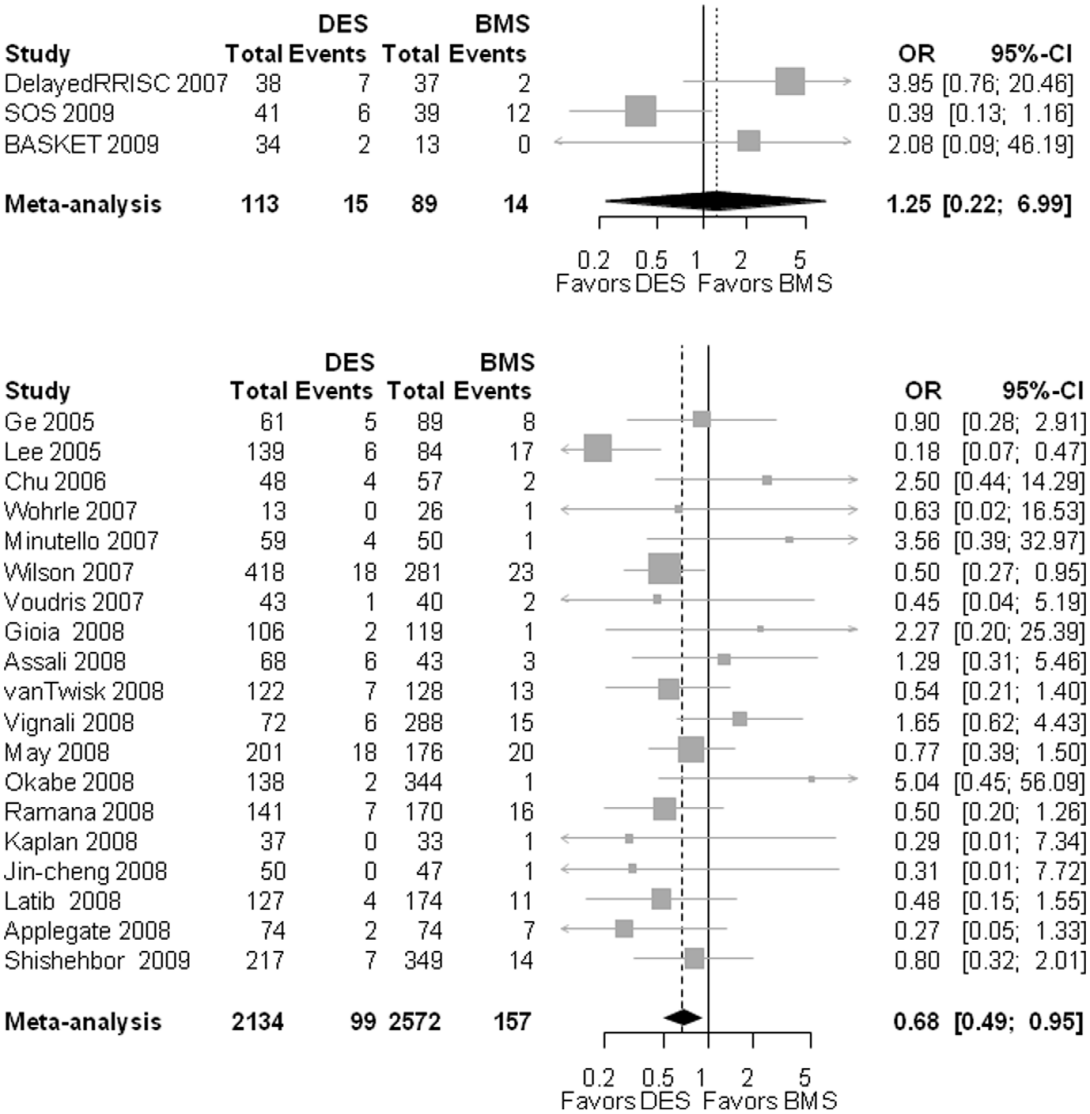
The Forest plot of odds ratios (OR) of myocardial infarction. Sizes of data markers are proportional to the weight of each study in the meta-analysis. Horizontal bars, 95% CI. Observational = observational, non-randomized controlled studies; DES = drug-eluting stent; BMS = bare metal stent; RCT = randomized controlled trials.

In the 3 RCT, infarction occurred in 13.7% [7.0–21.7%] of patients after DES implantation compared to 11.1% [0.5–33.1%] after BMS implantation. The OR for RCT exclusively was 1.25 [0.22–6.99]; p = 0.250; heterogeneity: I^2^ = 64.8%; p = 0.058) (Figure 3). In the observation studies, the OR for myocardial infarction after DES compared to DES was found to be 0.68 [0.49–0.95]; p = 0.023; heterogeneity: I^2^ = 23%; p = 0.183).

#### Stent thrombosis

**Figure 4:**
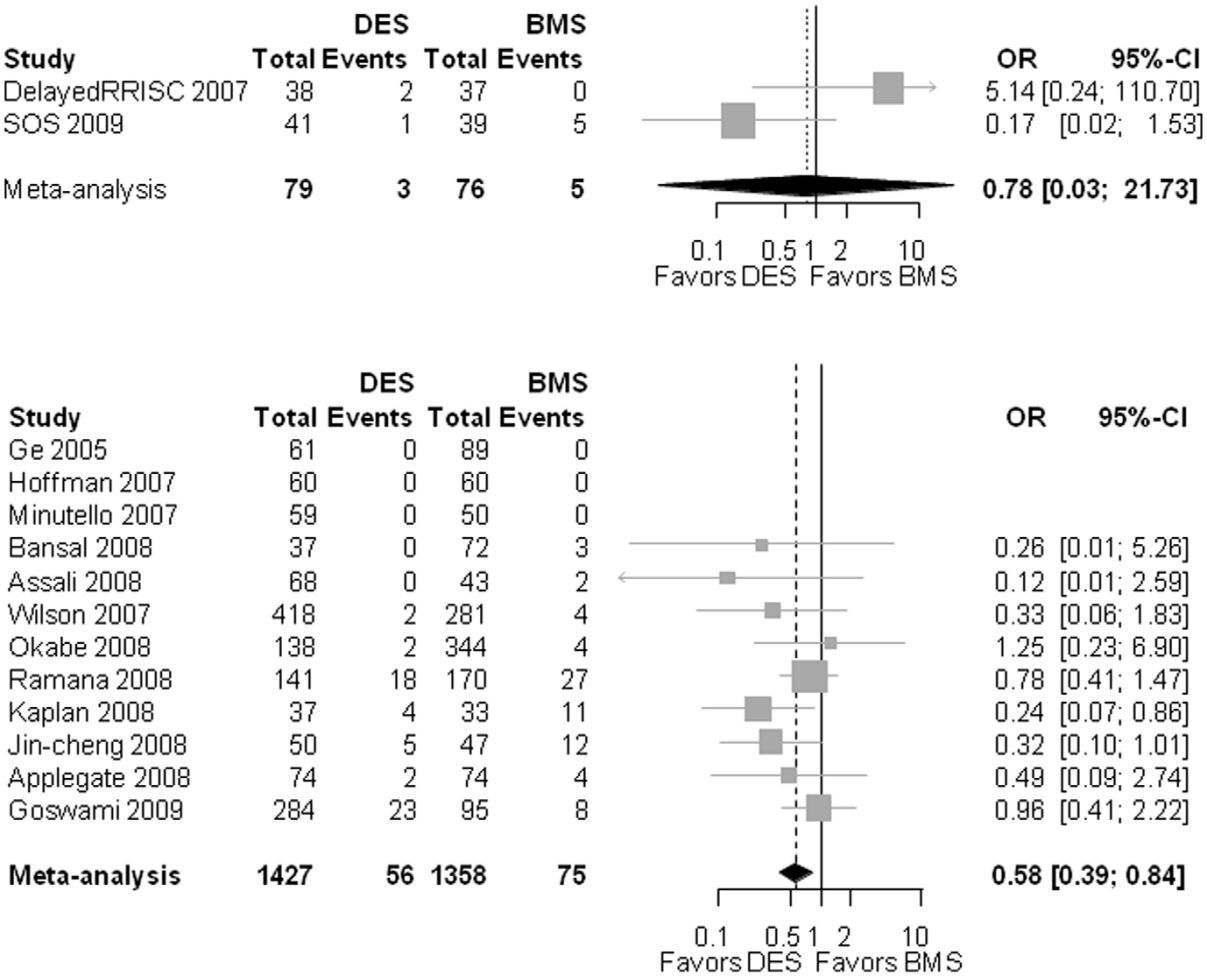
The Forest plot of odds ratios (OR) of stent thrombosis (ST). Sizes of data markers are proportional to the weight of each study in the meta-analysis. Horizontal bars, 95% CI. Observational = observational, non-randomized controlled studies; DES = drug-eluting stent; BMS = bare metal stent; RCT = randomized controlled trials.

In the 2 RCT reporting on this endpoint, the OR for DES compared to BMS was 0.78 [.03–21.73], p = 0.885; heterogeneity: I^2^ = 68.2%; p = 0.076 (Figure 4). The OR for the observational studies was in favor of DES (0.58 [0.38–0.84]; p<0.001; heterogeneity: I^2^ = 0%; p = 0.485).

#### Mortality

**Figure 5:**
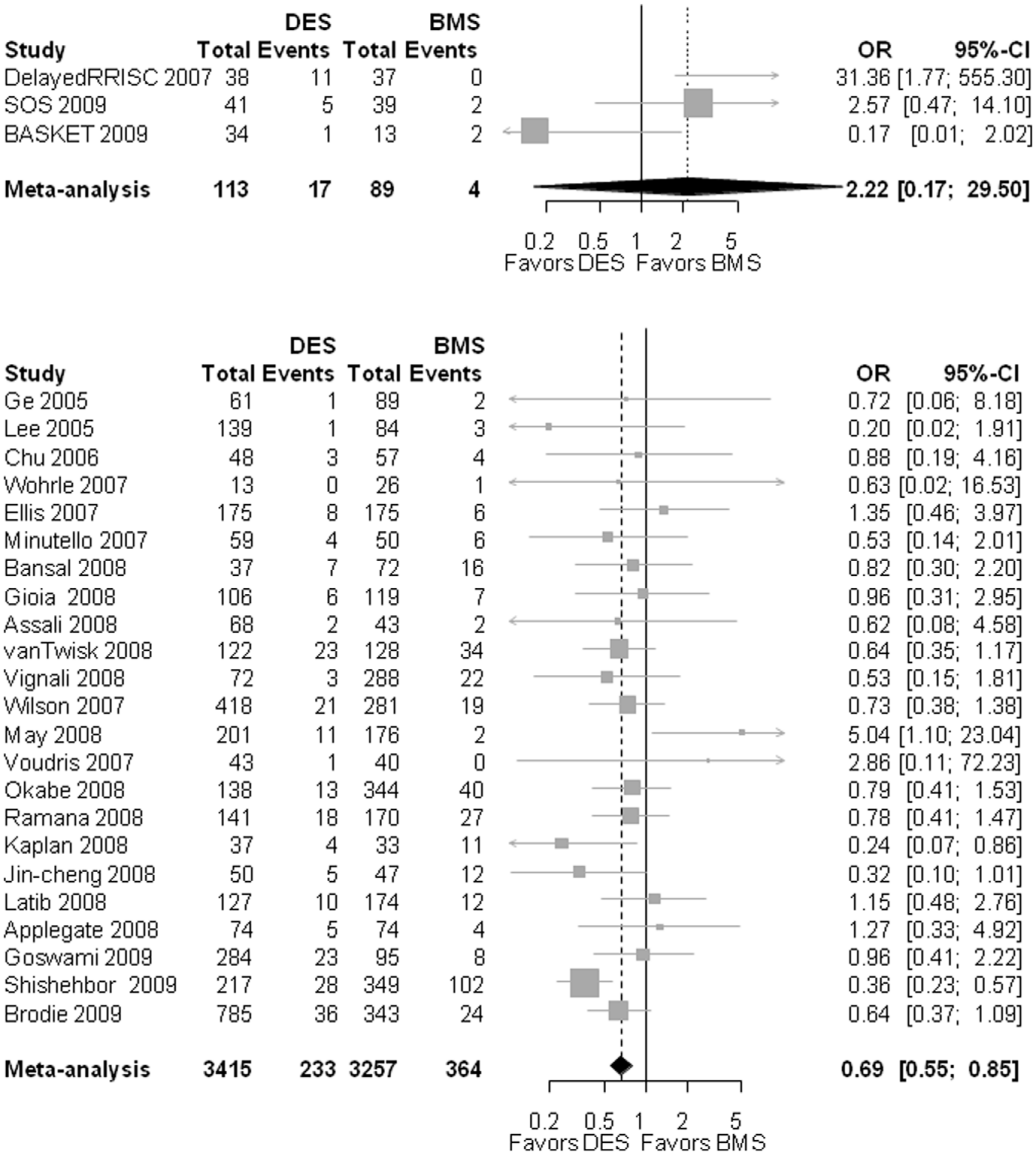
The Forest plot of odds ratios (OR) of mortality. Sizes of data markers are proportional to the weight of each study in the meta-analysis. Horizontal bars, 95% CI. Observational = observational, non-randomized controlled studies; DES = drug-eluting stent; BMS = bare metal stent; RCT = randomized controlled trials.

For the 3 RCT, the OR for mortality between DES and BMS was 2.22 (0.17 – 29.50; p = 0.546; heterogeneity: I^2^ = 75.8%; p = 0.019). For the observational studies, the OR for mortality for DES compared to BMS was 0.69 [0.55–0.85]; p<0.001; heterogeneity: I^2^ = 19%; p = 0.202] (Figure 5).

### Sensitivity and bias analyses

At the scientific meeting of the American College of Cardiology ACC 2010, longer term follow up data on the SOS trial have been presented (median 29 months). [44] The inclusion of these longer term data resulted in similar findings:

For the three RCT and in line with the above described results, the TVR was lower in the DES groups (OR 0.40 [0.16–0.96], p = 0.0405, heterogeneity: I^2^ = 48%, p = 0.147). The myocardial infarction risk was again not significantly different between the two groups (OR 1.02 [0.11–9.65], p = 0.986; heterogeneity: I^2^ = 79%, p = 0.008). There was no difference in the ST risk (OR 0.71 [0.02–24.25], p = 0.849). The mortality rate was not significantly different between the groups when considering the longer term data of the SOS trial (OR 1.92 [0.17–21.33], p = 0.597, heterogeneity: I^2^ = 75%, p = 0.019).

Also, we assessed for publication bias of RCT data by the Egger test and by visual assessment of a funnel plot. For the endpoint TVR, the Egger test revealed a p value of 0.228.

### Heterogeneity assessment

In order to explain the heterogeneity of results for the primary endpoint among the included studies (RCT and observational studies), we evaluated the influence of the type of drug eluting stent (sirolimus eluting stents (SES) versus paclitaxel eluting stents (PES). The effect in PES appeared more pronounced in the included studies compared to SES stents (OR 0.29 [0.14–0.63] versus 0.56 [0.38–0.81]), however, this stent type effect was not found to be statistically significant (p = 0.142)(Figure S1). A second factor that appears to matter is the time effect. The 2 studies published in 2005 showed an overall odds ratio of 0.19 [0.10–034]in favor of DES, more recent studies from 2009 and 2010 showed lower overall benefit for DES (OR 0.78 [0.49–1.23] and OR 0.96 [0.41–2.22], respectively) (Figure S2).

As a third factor, study size appeared to influence the effect size. According to Egger's test, there is a significant “small study effect”, smaller studies reported more pronounced superiority of DES compared to larger studies (bias estimate Egger's test: −1.71, standard error 0.77, slope 0.24, p = 0.034, Figure S3).

The fourth tested covariate was duration of follow up, which was not found to relevantly influence outcome (data not shown).

## Discussion

In this meta-analysis of 29 studies (3 RCT and 26 observational studies) including 7549 patients, DES were superior to BMS with regard to TVR while no difference was found in risk for myocardial infarction or stent thrombosis in the RCT. The observational studies revealed a reduced risk for stent thrombosis and mortality risk for DES and a trend toward decreased infarction. However, these latter differences may at least partially be explained by selection bias.

Preventing target vessel revascularization with DES may be of particular importance in SVG, in which stent failure often presents as an acute coronary syndrome, or with complete SVG lumen occlusion.[45]

Although restenosis rates are markedly higher in SVG compared with native vessels, classically, BMS is the treatment of choice for SVG stenoses[8] while this setting is regarded an off-label use for DES in the U.S. However, DES are commonly used in various clinical settings to treat native coronary artery lesions and have been shown to reduce restenosis rates, especially in patients with higher risk for restenosis (diabetes mellitus, small vessels etc.). Saphenous vein graft stenting clearly represents a higher risk setting. Thus, DES are nowadays increasingly being used off-label to treat SVG stenoses, there are limited safety and efficacy data available in this setting. On the other hand, there have been even increased concerns and data suggesting that the effect of DES may be attenuated by the different biological properties of vein grafts or that DES may even be harmful.[9], [19]

Due to a lack of clear evidence of optimal stent choice in saphenous vein grafts with only few small randomized trials, the optimal stent choice has been highly controversial over many years and this debate is still ongoing. The use of drug-eluting stents has decreased dramatically in many centers after data about increased risk for stent thrombosis and other negative aspects of coated stents have been published.[46], [47], [48]

### Heterogeneity among studies

Randomized controlled trials: The most significant reduction in the primary endpoint TVR was found in the BASKET trial, [7] the least effect in the Delayed RRISC trial [19] while the effect in the SOS trial [8] was somewhat in between. One difference of potential importance is the type of drug eluting stent that was used. While RRISC used sirolimus eluting stents (SES), SOS used paclitaxel eluting stents (PES), in the BASKET trial, both stent types were used. There may be a difference in the effectiveness of these substances when used in vein grafts. Another probably important difference among the studies is the difference in follow up interval. In the Delayed RRISC study, median follow up duration was 30.5–32 months, for BASKET and SOS it was 18 months. An interim analysis with shorter term results of the RRISC trial at 6 months follow up showed an impressive relative risk reduction of 0.19 (95% CI 0.05 to 0.83) for BMS, [6] while at 30.5–32 months, the relative risk reduction was only 0.90 [0.49–1.65]. [19] There seems to be a more pronounced early benefit while longer-term benefits seem less pronounced as described above. Recent observational data also suggested a late “catch-up” phenomenon regarding TVR with a clear benefit for DES in the first year but similar longer term results.[49] It seems plausible that, after the coating drug has completely eluted, the beneficial effect of DES compared to BMS decreases. Due to the different biological properties of saphenous vein grafts, this late “catch up” phenomenon may be more pronounced than in native vessels. Moreover, the RRISC delayed trial found that patients with SES had higher mortality rates than their BMS counterparts and similar rates of TVR on the long term. [6], [19].

Overall (observational studies and RCT): While the study heterogeneity in the RCT was limited, the results between the studies differed significantly when also considering observational studies. Several factors may significantly contribute to this heterogeneity:

First, type of drug eluting stent: The effect in PES appeared more pronounced in the included studies compared to SES stents (OR 0.294 [0.138–0.628] versus 0.555 [0.380–0.811]). This is in line with the finding in the RCT as described above, where the SOS trial using PES showed a more pronounced effect. Thus, the coating drug may play a significant role. While SES have proven to be more effective in native coronary vessels,[50] PES may be more effective in vein grafts (Figure S1). Second, time effect: While early studies (published in 2005) show a very impressive effect of DES, later studies found less benefit for DES compared to BMS. This may be related to changes of the tested stents themselves, it may also be related to other time-dependent co-factors. An improvement of the comparator (BMS) over time could have resulted in smaller differences compared to DES. Of note, the medical co-treatments have changes as well over time (improvement in lipid-lowering treatment, anti-platelet therapy etc.), leading to a general reduction in need for revascularizations and therefore, less significant differences between the two stents in this regard (Figure S2). We have observed a similar time effect in another setting of stenting, i.e., in carotid artery stenosis, where differences compared with the comparator treatment (carotid endarterectomy) relevantly decreased over time.[51] Third, study size: According to Egger's test, there seems to be a significant “small study effect”, small studies showed more pronounced effects than larger studies which may be due to publication bias (Egger's test p value = 0.034). This is illustrated in the linear regression plot of normalized effect sizes against precision (reciprocal of the standard error of the estimate) (Figure S3). Fourth, there is a wide range of follow up duration among the included studies. While this may have influenced the findings in the RCT, it does not appear to have relevantly influenced the findings overall but probably adds to the overall heterogeneity as well.

### Limitations

The main limitation of this study is the small number of RCT available for inclusion. Furthermore, each of the 3 RCT was rather small. [6], [7], [8] Therefore, the statistical power of this analysis is small and the primary endpoint, TVR, did not quite reach statistical significance. Thus, it is too early to draw strong conclusions based on these limited available data. However, the meta-analysis of the observational studies are reflecting the “real-world ” and further support the conclusion but observational data are of course prone to bias toward patient selection. [23] It also has to be noted that evaluation of publication bias cannot be done in a robust manner with such few data points, the statistical power of the Egger's test to suspect publication bias is very limited here.

We have to acknowledge that even our pooled analysis is very limited in statistical power and the results showed only a borderline significance for TVR. On the other hand, the observational studies in this meta-analysis support that DES may be beneficial regarding TVR in SVG. Observational data are, of course, prone to bias due to non-random treatment allocation. Further, it must be noted that a majority of the studies had a short follow-up period (6–12 months).

### Conclusion

The use of DES may be superior to the use BMS for treatment of SVG with regard to TVR but this finding is mainly based on observational data while the analysis based on 3 small RCT did not reach statistical significance. However, the finding is supported by a significant reduction in TVR seen in observational studies. Based on the RCT data, there are probably no major differences in safety endpoints such as myocardial infarction, stent thrombosis or mortality while observational data indicate lower risk for death, stent thrombosis and myocardial infarction for the DES group, a finding that may reflect selection bias in these observational studies or a true finding that was not detected in the RCT due to lack of statistical power.

## Supporting Information

Figure S1 The Forest plot of odds ratios (OR) of target vessel revascularization (TVR), stratified by stent type. Horizontal bars, 95% CI. DES = drug-eluting stent; BMS = bare metal stent; RCT = randomized controlled trials.
(0.02 MB TIF)

Figure S2 The Forest plot of odds ratios (OR) of target vessel revascularization (TVR), stratified by publication year. Horizontal bars, 95% CI. DES = drug-eluting stent; BMS = bare metal stent; RCT = randomized controlled trials.
(0.02 MB TIF)

Figure S3 Effect of study size. The linear regression of standardized effect size (regarding target vessel revascularization) versus inverse of the standard error of the effect size ( =  precision), which generally speaking reflects study size.
(0.03 MB TIF)

Table S1 Study quality of included randomized controlled trials according to the Jadad score.
(0.04 MB DOC)

File S1 Study plan and abstract form.
(0.03 MB DOC)
