# Supplementary figures and images for: Drug-Eluting versus Bare-Metal Stent for Treatment of Saphenous Vein Grafts: A Meta-Analysis
Source: PLoS One. 2010 Jun 10;5(6):e11040. doi: 10.1371/journal.pone.0011040 (PMC2883580; doi:10.1371/journal.pone.0011040)

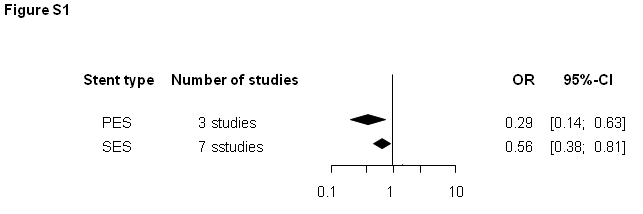

Supplement: Figure S1 — The Forest plot of odds ratios (OR) of target vessel revascularization (TVR), stratified by stent type. Horizontal bars, 95% CI. DES = drug-eluting stent; BMS = bare metal stent; RCT = randomized controlled trials. (0.02 MB TIF) [file pone.0011040.s001.tif]

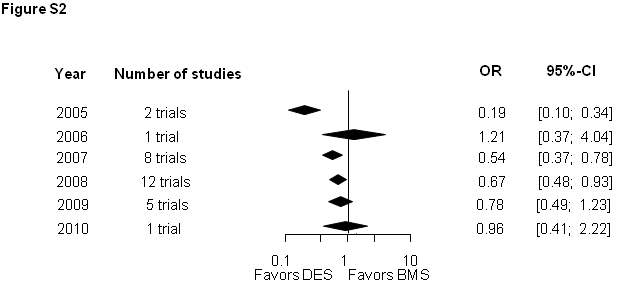

Supplement: Figure S2 — The Forest plot of odds ratios (OR) of target vessel revascularization (TVR), stratified by publication year. Horizontal bars, 95% CI. DES = drug-eluting stent; BMS = bare metal stent; RCT = randomized controlled trials. (0.02 MB TIF) [file pone.0011040.s002.tif]

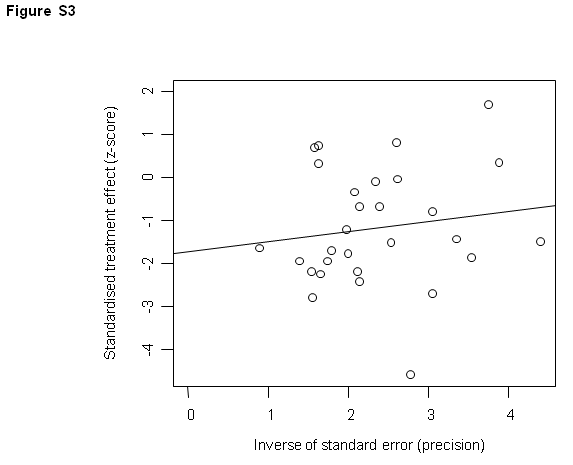

Supplement: Figure S3 — Effect of study size. The linear regression of standardized effect size (regarding target vessel revascularization) versus inverse of the standard error of the effect size ( = precision), which generally speaking reflects study size. (0.03 MB TIF) [file pone.0011040.s003.tif]
