# Supplementary material for: Drug-Eluting versus Bare-Metal Stent for Treatment of Saphenous Vein Grafts: A Meta-Analysis
Source: PLoS One. 2010 Jun 10;5(6):e11040. doi: 10.1371/journal.pone.0011040 (PMC2883580; doi:10.1371/journal.pone.0011040)
Supplement: Table S1 — Study quality of included randomized controlled trials according to the Jadad score. (0.04 MB DOC) [file pone.0011040.s004.doc]

| Table S1: Study quality of individual randomized trials (Jadad score) | | | | | | | |
| --- | --- | --- | --- | --- | --- | --- | --- |
| **Study** | **Randomized** | **Appropriate randomization** | **Double blind** | **Appropriate blinding** | **Drop outs appropriately declared** | **Score** | **Quality** |
| BASKET | yes | yes | no | na | yes | 3 | sufficient |
| Delayed RRISC | yes | yes | no | na | yes | 3 | sufficient |
| SOS | yes | yes | no | na | yes | 3 | sufficient |
| na: not applicable |  |  |  |  |  |  |  |
