# Supplementary material for: Drug-Eluting versus Bare-Metal Stent for Treatment of Saphenous Vein Grafts: A Meta-Analysis
Source: PLoS One. 2010 Jun 10;5(6):e11040. doi: 10.1371/journal.pone.0011040 (PMC2883580; doi:10.1371/journal.pone.0011040)
Supplement: File S1 — Study plan and abstract form. (0.03 MB DOC) [file pone.0011040.s005.doc]

Primary focus: randomized controlled trials comparing DES and BMS in patients with saphenous vein graft (SVG) stenosis.

Secondary focus: non-randomized controlled studies comparing DES and BMS in patients with saphenous vein graft (SVG) stenosis.

Endpoints:

Primary outcome of interest: target vessel revascularization TVR (or target lesion revascularization, TLR)

Secondary outcomes:myocardial infarction, stent thrombosis, death.

Data sourses:

EMBASE, MEDLINE, Cochrane Central Register of Controlled Trials, International Pharmaceutical Abstracts database, ISI Web of Science, and google scholar.

Starting point: January 1, 2002

Scientific meetings, starting point January 1, 2006:

American College of Cardiology, European Society of Cardiology, the Transcatheter Cardiovascular Therapeutics, and the American Heart Association.

Search terms: **“**saphenous vein graft**”,** **“**bare-metal stent**”,** **“**drug-eluting stent**”,** **“**paclitaxel-eluting stent”, **“**sirolimus-eluting stent**” , “**everolimus-eluting stent**”** ,” zatarolimus-eluting stent”,“stents**”**.

**Data Extraction Form**

**General**

Study Number ___________ Reviewer ____________

Author, Year, Journal _____________________________________________

**Methods**

Study population (n) _________________________________________

Randomized: y_____ n______

If yes, appropriate randomization: y_____ n______

Blinded outcome assessment: y_____ n______

Appropriate blinding: y_____ n______

Drop outs appropriately declared: y_____ n______

**Results**

Final population of DES group: ______ Final population of control group: _______

Mean (or median) age for each group: DES _______ control ________

Mean or median duration of follow up:

DES _______ control ________

Stent type:

DES _______ control ________

Vein graft age:

DES _______ control ________

Protection device used:

DES _______ control ________

Number of TVR (or TLR if TVR not given):

DES _______ control ________

Number of death:

DES _______ control ________

Number of stent thromboses:

DES _______ control ________

Number of myocardial infarctions:

DES _______ control ________
